# *Echinococcus granulosus*: Cure for Cancer Revisited

**DOI:** 10.3389/fmed.2018.00060

**Published:** 2018-03-12

**Authors:** Shiwanthi L. Ranasinghe, Donald P. McManus

**Affiliations:** ^1^Molecular Parasitology Laboratory, Department of Immunology, QIMR Berghofer Medical Research Institute, Brisbane, QLD, Australia

**Keywords:** *Echinococcus granulosus*, cancer therapy, Kunitz type protease inhibitor, antibody-mediated response, *Echinococcus* antigens

## Abstract

Whereas a number of parasites are well recognized risk factors for a number of different cancers in mammalian hosts, there is limited information on the ability of parasitic organisms to induce anticancer effects. There are conflicting reports that echinococcosis, caused by the canine tapeworm *Echinococcus granulosus*, can decrease or increase cancer risk. This review considers both indirect anticancer effects as the result of adaptive immunity generated against certain echinococcal antigens and the direct effect of molecules released by *E*. *granulosus* whose activity directly inhibits cancer cell migration and growth. In conclusion, *E*. *granulosus* probably secretes molecules that can be developed as anticancer therapeutics in future.

## Introduction

There is increasing evidence that some infectious agents induce antitumor activity against different types of cancers (1). Experiments *in vitro* have shown that certain parasites including the protozoans *Trypanosoma cruzi* (2), *Toxoplasma gondii* (3), and *Acanthamoeba castellanii* (4) and the helminths *Echinococcus granulosus* (5) and *Strongyloides stercoralis* (6) exhibit anticancer activities. However, there are conflicting reports in the literature that the canine tapeworm (Phylum Cestoda) *E*. *granulosus*, the cause of human cystic hydatid disease (echinococcosis) in many parts of the world is able to reduce cancer growth (7–9). A significantly lower prevalence of cancer was reported in patients with hydatid disease in a large retrospective study in Turkey (7). In direct contrast, a pilot retrospective study carried out in Cyprus indicated that echinococcosis may increase cancer risk in patients (10). In the main, there is more evidence to support the concept that *E*. *granulosus* reduces cancer growth. This can be by a direct effect or indirectly by the development of immunity against common antigens associated with cancer and echinococcosis.

## *Echinococcus* and Adaptive Immunity

In an early study, antigenic similarity was reported between pulmonary carcinoma and hydatid cyst fluid (11). However, common antigens present in *E*. *granulosus* and some tumor types were thought to modulate host immune responses inducing anticancer activity (12). Cancers and parasites share similar properties in that both express mucin-type O-glycans, which are not usually found on healthy cell surfaces (13, 14). O-glycans present in cancer cells play key roles in metastasis, cell adhesion, and invasion (15). Cancer-associated O-glycosylated Tn (α-*N*-acetylgalactosamine-O-serine/threonine) antigens have been detected in both larval and adult *E*. *granulosus* worm extracts with most activity recorded in the adult excretory/secretory (ES) products (16). More recently, antigens, which may be mucin type O-glycans, have been identified in hydatid cyst fluid, in laminated and germinal layers, and in the ES products of hydatid cyst protoscoleces (12). Immunological cross-reactivity of hydatid cyst fluid antigens with sera from cancer patients has been reported to be at an unusually higher level than in sera from healthy individuals (17). Therefore, antibody-mediated immune responses induced by these Tn antigens in echinococcosis patients are considered to induce immunity against cancer growth (12). Mucin-like peptides from *E*. *granulosus* (Egmuc) have been shown to induce an increase in activated natural killer (NK) cells in the spleens of immunized mice in a process mediated by soluble dendritic cell-derived factors (18). *In vivo* primed-splenocytes with Egmuc peptides induce pancreatic tumor cell cytotoxicity *in vitro*. However Egmuc-specific antibodies hardly recognized tumor derived antigens; therefore, the anticancer effects of Egmuc are probably due to its stimulating NK cell activation and inducing a Th1-like response (18). In contrast to control sera from people with no history of echinococcosis, sera from patients with hydatid disease have a cytotoxic effect on human lung small cell carcinoma cells (19) providing additional evidence of antibody-mediated immunity against cancer. Furthermore, 40% of mice vaccinated with hydatid cyst fluid were shown to induce tumor regression in a colon cancer model and also induced an adaptive immune response against tumor re-challenge (20).

An experimental breast cancer model study, where rats had induced mammary carcinogenesis, showed that animals 20 days post echinococcosis infection showed reduced tumor growth compared with uninfected rats (21). Overall, these studies suggest that some *E*. *granulosus* antigens can induce memory cell formation to attack similar cancer-associated antigens. A proteomics study identified two proteins, mortalin (GRP75) and creatine kinase M-type, as being present both in *E*. *granulosus* and in colon cancer (20), and it has been reported that intratumoral and intraperitoneal injections of anti-GRP75 antibodies suppressed tumor growth (22). In contrast, a monoclonal antibody developed against a 40 kDa band in *E*. *granulosus* hydatid cyst fluid, which bound serum from breast cancer patients, had no significant effect on the growth of breast cancer cells *in vitro* (23).

Experiments *in vitro* have shown that neutrophils are actively involved in the killing of *E*. *granulosus* oncospheres (eggs; the infective stages to humans and ungulates) indicating an antibody-dependent, cell mediated response (24). However, in progressed malignancy, a substantial antitumor immune response is needed to eliminate cancer cells (25). A hallmark of immunotherapy is long-term memory of the adaptive immune response (26). This may be an explanation why echinococcosis patients in endemic areas develop resistance to cancer (7). In direct contrast, injection of 4T1 mouse breast cancer cells into mice with experimental secondary echinococcosis resulted in an increased level of cancer metastasis in the liver, which was associated with a reduced Th1 immune response (9).

Overall, many studies are in favor of using *E*. *granulosus* antigens in cancer therapy. However more research is needed to identify the specific molecules of this parasite which reduce the cancer risk and/or can act as potential future treatments.

## Cancer Killing Immune Responses Against Echinococcosis

Cancer cells are able to evade host immunity through various mechanisms, eventually establishing a relationship that mimics a chronic infection (27). Following their initial recognition, Th-1 polarized lymphocytes activate cytotoxic T cells and macrophages to destroy cancer cells (28). There is evidence showing that a Th-1 polarized response is protective against several cancers whereas patients with a polarized Th-2 response have poor prognosis in breast, lung, colorectal, and pancreatic cancers (29). In the early stages of echinococcosis, a Th1 immune response dominates, but during cyst establishment and growth, there is a switch to a Th-2 response, which is beneficial to the parasite for survival (30). When the hydatid cyst is either dying or dead, the Th2 response wanes rapidly allowing a Th1 response to take over (30).

As the recognition of specific tumor-associated antigens is the crucial step initiating an antitumor immune response (31), exposure to cancer-like antigens expressed by the *Echinococcus* parasites can stimulate such an anti-cancer response.

## Direct Cancer Cell Killing by *Echinococcus*

Apart from generating antibody-mediated immunity, there is some evidence suggesting that *Echinococcus* parasites can directly kill cancer cells. Hydatid cyst protoscoleces have been shown to inhibit the proliferation of baby hamster kidney fibroblasts and induce the death of fibrosarcoma cells *in vitro* (5), although the specific molecules involved are not known. In an *in vivo* study in C57BL/6 mice, treatment with hydatid cyst fluid concurrently with the injection of melanoma cells resulted in a reduction in tumor growth (32). However, it was not clear in this study whether the control mouse group also received alum as an adjuvant control either injected intraperitoneally or in to the tumor margin (32). A potential concern is that being an aluminium-based adjuvant, alum can selectively stimulate a Th2 immune response in mice (33) which might play a role in anti-cancer effects (34).

EgKI-1 is a recently identified potent Kunitz type protease inhibitor highly expressed in oncospheres of *E*. *granulosus* (35). EgKI-1 treatment inhibits the growth and migration of a variety of cancer cells *in vitro* by negatively affecting cell cycle progression causing apoptosis.^1^ Furthermore, EgKI-1 treatment significantly reduced tumor growth in a triple negative breast cancer model (see text footnote 1).

## Neutrophils and Echinococcosis

There is marked activity of cell-mediated immunity during the acute phase of echinococcosis including the infiltration of inflammatory cells, which mainly comprise neutrophils and macrophages (30). Proteases such as neutrophil elastase (NE), secreted by activated neutrophils, can digest foreign parasite bodies and induce neutrophil chemotaxis. As a potent NE inhibitor of the secretory type, EgKI-1 from the oncospheres of *E*. *granulosus* might protect this stage from the host immune system (35).

**Figure 1:**
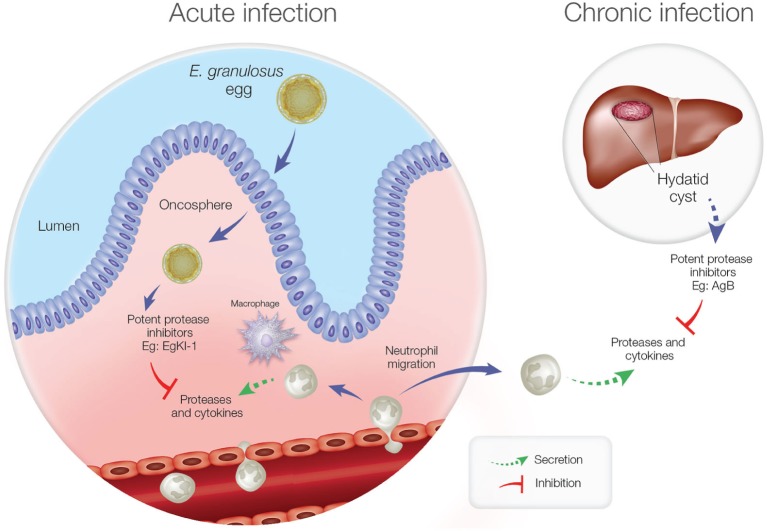
Neutrophil function during acute and chronic echinococcosis. In the acute stage, neutrophils and macrophages migrate to the intestinal mucosa to attack invading oncospheres. In the chronic stage, if hydatid cyst fluid leakage occurs from a ruptured cyst, neutrophils are attracted but antigen B inhibits neutrophil chemotaxis and neutrophil elastase to protect protoscoleces so they can develop into new cysts.

Furthermore, in the chronic stage of echinococcosis, if the hydatid cyst ruptures, neutrophils are attracted to kill the contained protoscoleces, and this might be a reason for the elevated expression of Antigen B (AgB) in hydatid cyst fluid (36). Being another potent protease inhibitor, AgB can significantly reduce neutrophil recruitment, thus delaying the potential killing of protoscoleces by neutrophils until the larvae can grow into larger cysts resulting in secondary echinococcosis. Inhibiting NE secretion and neutrophil chemotaxis is, therefore, important for *E*. *granulosus* survival during both the acute and chronic disease phases (Figure 1).

## Neutrophils and Cancer

**Figure 2:**
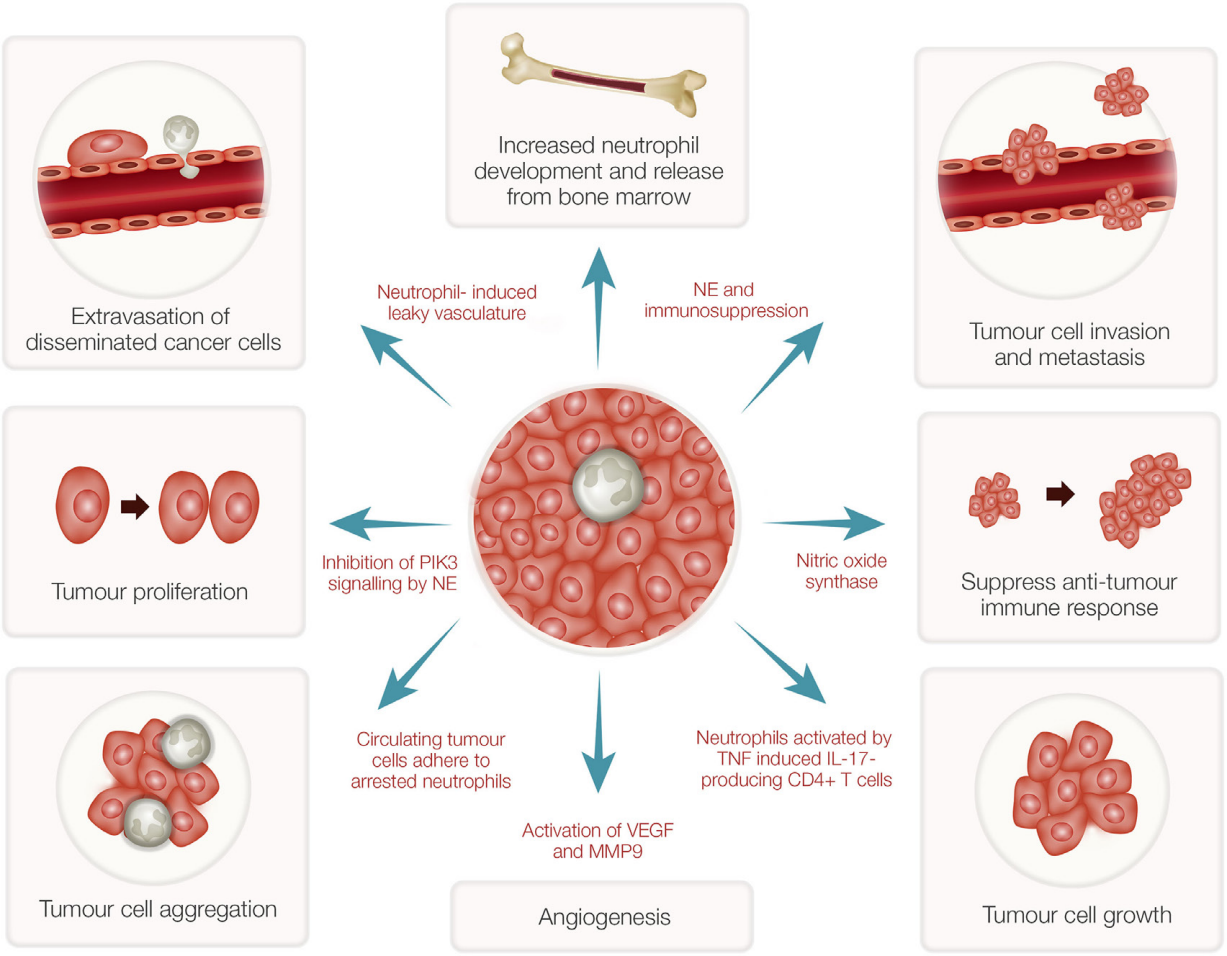
Tumor-associated neutrophils (TANs) and cancer growth. TANs can stimulate bone marrow to develop and release more neutrophils. Reactive oxygen species and proteases secreted from activated neutrophils can induce epithelial damage and subsequent tumor promoting inflammation. Nitric oxide synthase expressed by neutrophils suppresses CD8^+^ T-cell-mediated antitumor immune responses thus stimulating tumor progression. Increased neutrophils can induce leaky vasculature facilitating extravasation of disseminated cancer cells and aggregation leading to tumor metastasis. Neutrophil elastase (NE), secreted by activated neutrophils, can inhibit phosphoinositide 3-kinase (PIK3) signaling, and suppress immune reactions leading to tumor growth and proliferation. Further, neutrophils induce angiogenesis by activation of vascular endothelial growth factor (VEGF) mediated by matrix metalloproteinase 9 (MMP9).

While neutrophils play a major role in host defense, these cells have both pro- and antitumor effects in cancer patients (37). Many subjects with advanced cancers show high numbers of neutrophils in their blood (38) even though the precise mechanisms involved are unknown. Recent evidence has indicated that neutrophils in the tumor microenvironment actively contribute to tumor growth initiation, progression, metastasis, and angiogenesis (39–41) (Figure 2). Consequently, potent NE inhibitors have been tested as anticancer therapeutics (42). As indicated above, as a potent NE inhibitor, the EgKI-1 protein exhibits anticancer effects both *in vitro* and *in vivo* (see text footnote 1).

## *Echinococcus* Antigens in Cancer Therapy

As referred to earlier, there is evidence suggesting that some *Echinococcus* antigens have the capacity to induce antibody-mediated immunity, which can induce non-specific immunity against certain cancer types, whereas the EgKI-1 protein secreted by *E*. *granulosus* oncospheres is able to kill cancer cells directly. As AgB is also a potent NE inhibitor, it would be profitable to investigate whether it can also kill cancer cells. However, apart from NE inhibition, other mechanisms and/or molecules, which interact with EgKI-1 causing the observed anticancer effects *in vivo*, are likely involved and still need to be investigated. Furthermore, bioavailability and serum clearance can determine how released molecules from a parasite such as *Echinococcus* act *in vivo* although it is known that the “excretory/secretory” products from helminth worms can circulate in the body through the lymph or blood (28).

## Conclusion

Scrutiny of the available literature suggests that certain *Echinococcus* antigens can generate adaptive immunity against cancer. Moreover EgKI-1, which is secreted by *E*. *granulosus*, shows direct anticancer effects. Therefore, this canine tapeworm may actually provide some hope as a potential cure against some forms of cancer. Additional studies are now required to progress this research further and to identify additional specific proteins secreted by this tapeworm for application in future anticancer therapy.

## Author Contributions

SR drafted the manuscript and DM critically evaluated and edited the manuscript.

## Conflict of Interest Statement

The authors declare that the research was conducted in the absence of any commercial or financial relationships that could be construed as a potential conflict of interest.

## Footnotes

^1^Ranasinghe SL, Boyle GM, Fischer K, Potriquet J, Mulvenna JP, McManus DP. EgKI-1, a promising anti-cancer therapeutic from the canine tapeworm *Echinococcus granulosus*. *Sci Rep*. (Under Review).
